# Treatment of Chronic Hepatitis C in the Aged – Does It Impact Life Expectancy? A Decision Analysis

**DOI:** 10.1371/journal.pone.0157832

**Published:** 2016-07-13

**Authors:** Yaakov Maor, Stephen D. H. Malnick, Ehud Melzer, Moshe Leshno

**Affiliations:** 1 Institute of Gastroenterology and Hepatology, Kaplan Medical Center, Rehovot, Israel; 2 Department of Internal Medicine C, Kaplan Medical Center, Rehovot, Israel; 3 Faculty of Management and Sackler Faculty of Medicine, Tel Aviv University, Tel Aviv, Israel; The Chinese University of Hong Kong, HONG KONG

## Abstract

**Background and Aims:**

Recent studies have demonstrated that the efficacy of interferon-free direct-acting antiviral agents (DAAs) in patients over 70 is similar to that of younger age groups. Evidence continues to mount that life expectancy (LE) increases with successful treatment of hepatitis C (HCV) patients with advanced fibrosis. The evidence in older people is more limited. Our aim was to estimate the life year (LY) and quality-adjusted life year (QALY) gained by treatment of naïve patients with HCV as a function of patient's age and fibrosis stage.

**Methods:**

We constructed a Markov model of HCV progression toward advanced liver disease. The primary outcome was LY and QALY saved. The model and the sustained virological response of HCV infected subjects treated with a fixed-dose combination of the NS5B polymerase inhibitor Sofosbuvir and the NS5A replication complex inhibitor Ledipasvir were based on the published literature and expert opinion.

**Results:**

Generally, both the number of LY gained and QALY gained gradually decreased with advancing age but the rate of decline was slower with more advanced fibrosis stage. For patients with fibrosis stage F1, F2 and F3, LY gained dropped below six months if treated by the age of 55, 65 or 70 years, respectively, while for a patient with fibrosis stage F4, the gain was one LY if treated by the age of 75. The QALY gained for treated over untreated elderly were reasonably high even for those treated at early fibrosis stage.

**Conclusions:**

There is a significant life expectancy benefit to HCV treatment in patients up to age 75 with advanced-stage fibrosis.

## Introduction

Hepatitis C (HCV) affects about 170 million people worldwide and is a leading cause of cirrhosis and hepatic insufficiency, and a reason for liver transplantation. In addition it accounts for more than 50% of incident hepatocellular carcinoma (HCC). The U.S. Centers for Disease Control and Prevention (CDC) and the U.S. Preventive Services Task Force (USPSTF) recently issued their recommendation for one-time testing of adults born during 1945–1965 (baby boomers) for HCV without prior ascertainment of HCV risk [1]. These recommendations, which are based on the finding that the members of this cohort, many of whom are now approaching 70, account for 76.5% of those with HCV antibodies in the US [1], led to the development of a multicohort natural history model for predicting disease outcomes and benefits of therapy [2]. The model projected a decline in the prevalence of HCV by 2030. However, it also predicted that the proportion of cases with advanced fibrosis will continue to rise during the next two decades, with the number of cases of cirrhosis and hepatic decompensation peaking after the year 2020. The study further predicted that the age of those with cirrhosis and its complications will continue to rise, with those aged 60 to 80 being most affected. As this age group overlaps the 1945–1965 birth cohorts, more advanced HCV can be seen as becoming a serious problem for the elderly.

In previous studies reported in the literature, the older population was largely excluded from the pivotal phase III registration trials of the first generation protease inhibitors and of interferon-free direct-acting antivirals (DAAs) [3–9]. Therefore, there are no guidelines for treatment of the elderly, defined as 70 years and older, a definition that is largely driven by the age limit in the major phase III trials. Recent observational studies have demonstrated that the efficacy of the first generation protease inhibitor-based regimens in patients over 65 is similar to that for younger age groups, though adverse effects are more frequent [10]. Likewise, sub-group analysis on a small number of elderly patients included in the registration DAA trials show comparable efficacy, with a sustained virological response (SVR) exceeding 90% [7–9]. Although these regimens have a favorable safety profile they are costly, a consideration that may be prohibitive particularly in those parts of the world with a high prevalence of HCV.

Recently, it has been shown that the beneficial effects of SVR also result in reduced all-cause mortality in the high-risk population of patients with chronic HCV infection and severe hepatic fibrosis [11–13]. The strongest evidence on the association between SVR and overall survival is a large Veterans Affairs cohort study that found SVR to be associated with a 30% to 50% reduction in mortality risk, after adjustment for many confounders [14]. As the median age of patients included in these studies was the late forties to the early fifties, the question whether elderly patients would actually benefit from HCV treatment with improved life expectancy (LE) remains open.

Our objective was to compare the estimated life years (LY) and quality-adjusted life years (QALY) using two strategies: treatment *vs*. no treatment of naïve patients with HCV as a function of patient's age and fibrosis stage in the U.S. population.

## Materials and Methods

### Model Construction

We constructed a Markov model of HCV natural history and progression toward advanced liver disease in order to assess LE and QALY. Markov models are employed to represent stochastic processes, that is, random processes that evolve over time. In a healthcare context, Markov models consider the patients in a discrete state of health, and the events represent the transition from one state to another. The possibility of modeling repetitive events and time dependence of probabilities and utilities associated permits an accurate representation of the evaluated clinical structure. The model of HCV natural history and the SVR of the currently approved DAA regimens were based on the literature. The model was developed in stages starting with a traditional bubble diagram of disease states that served as the basis for developing a more detailed mathematical model that followed the health state of HCV infected persons. We used cohort simulation with the following health states included: resolved infection, stage of fibrosis (F0 to F4 –cirrhosis) [15], liver failure, HCC, liver transplantation and liver-related deaths. Age-specific (non-liver) deaths were also included. In addition, we used Monte Carlo simulation to estimate the rate of HCC and liver transplantation.

### Data Sources

Table A in S1 File gives the assumptions of the Markov model. Transition rates for progressing from one fibrosis stage to another stage, according to the METAVIR classification (F0→F1; F1→F2; F2→F3; F3→F4), were largely based on the pooled rates from a meta-analysis reported by Thein and colleagues [16]. The progression of fibrosis is an essential factor in the Markov model analysis. It is well known that the progression of fibrosis may differ between individuals. Several reports [17–19] consistently demonstrate more rapid fibrosis progression rate for those over 50 than for those younger than 50. However, as data regarding the rate of fibrosis progression for HCV patients older than 70 are scarce, we considered a conservative linear mode of fibrosis progression in this age group. Likewise, compared with premenopausal women, postmenopausal women have more rapid fibrosis progression rate. We based our estimation of fibrosis rate on Thein et al [16], and used a base-case progression rate and a sensitivity analysis over a range of progression rates, which include rates applicable to postmenopausal women. Furthermore, the results of this study apply to a hypothetical average patient, but not to an individual person.

The SVR considered for the model was driven by two registration trials of the fixed-dose combination of the NS5B polymerase inhibitor Sofosbuvir and the NS5A replication complex inhibitor Ledipasvir in untreated HCV genotype 1 infection. We also included data derived from a sub-analysis of the treated elderly population included in these registration trials [7, 8]. This treatment combination was chosen since it represents the current acceptable SVR achieved by this and other approved all-oral DAA regimens for HCV genotype 1 in naïve patients.

Based on longitudinal studies in North America and Europe the annual risk of clinical decompensation, death or transplantation, and HCC has been estimated to be 6% (range, 4–8%), 3% (range, 2–6%), and 3% (range, 2–6%) per year, respectively [20–22]. We also assumed that the 1-year mortality was 5.5% in compensated and 20% in decompensated cirrhotics [23] HCC risk in those with F3 fibrosis was estimated to be 10% of that in cirrhosis. Age-specific (all-cause) deaths derived from standard mortality tables [24].

Regression of fibrosis following SVR was estimated from large cohorts of HCV patients attaining an SVR [25, 26]. These cohorts were evaluated for the evolvement of fibrosis stage using liver biopsy [25] or non-invasive measures of fibrosis [26]. The follow-up post-treatment period in the latest report was up to 10 years [26]. In the case of cirrhosis we estimated some regression of fibrosis in 50% of patients following an SVR. It was assumed that cirrhotic patients with SVR who had a regression of fibrosis had no subsequent hepatic decompensation. The risk of progression from cirrhosis to HCC after SVR was estimated to be 0.66% per year [26, 27], whereas patients with F3 stage fibrosis who attained an SVR were considered not likely to develop HCC (for all assumptions used to for the Markov model see Table A in S1 File). Background mortality was based on U.S. life tables.

The primary end-point of the study was number of LY and QALY gained for treated *vs*. untreated naïve patients with HCV. In general, we considered at least six months LY gained as life expectancy sufficient to warrant treatment.

### Sensitivity Analysis

Sensitivity analysis was performed to assess the extent to which the model’s calculations were affected by uncertainty in our assumptions. The ranges utilized in the sensitivity analysis were derived from the medical literature. Sensitivity analysis with tornado diagrams was utilized to rank the variables in the model with regard to their impact on LE (QALY). We then conducted one-way sensitivity analysis of the variables with high impact on LE (QALY).

Variables studied in the sensitivity analysis included: SVR (ranging from 0.8 to 0.99); annual rate of decompensation (ranging from 0.02 to 0.083); death rate for decompensated cirrhosis (ranging from 0.065 to 0.194); death rate for HCC (ranging from 0.33 to 0.86) and rate of HCC in decompensated cirrhosis (0.068 to 0.09).

## Results

### LY and QALY gained

LY gained for treated *vs*. untreated HCV patients ranged from 0.01 years for an 80-year-old with fibrosis level F1 to 10.20 years for a 40-year-old cirrhotic (Table 1). The actual life expectancy for treated *vs*. untreated HCV patients for each fibrosis stage is shown in Table B 1–4 in S1 File. Thus, for example, for a 65-year-old patient with fibrosis stage F3 the life expectancy is 81.420 years and 79.872 years for treated and untreated patients, respectively.

**Table 1:** Life year (LY) gained for treated *vs*. non-treated patients by age and initial fibrosis stage.

Age	F1	F2	F3	F4
**40**	2.84	6.09	9.85	10.20
**45**	1.89	4.36	7.47	8.35
**50**	1.19	2.98	5.45	6.64
**55**	0.70	1.92	3.80	5.10
**60**	0.38	1.16	2.50	3.76
**65**	0.19	0.65	1.55	2.64
**70**	0.08	0.33	0.89	1.74
**75**	0.03	0.15	0.47	1.07
**80**	0.01	0.06	0.23	0.62

**Fig 1:**
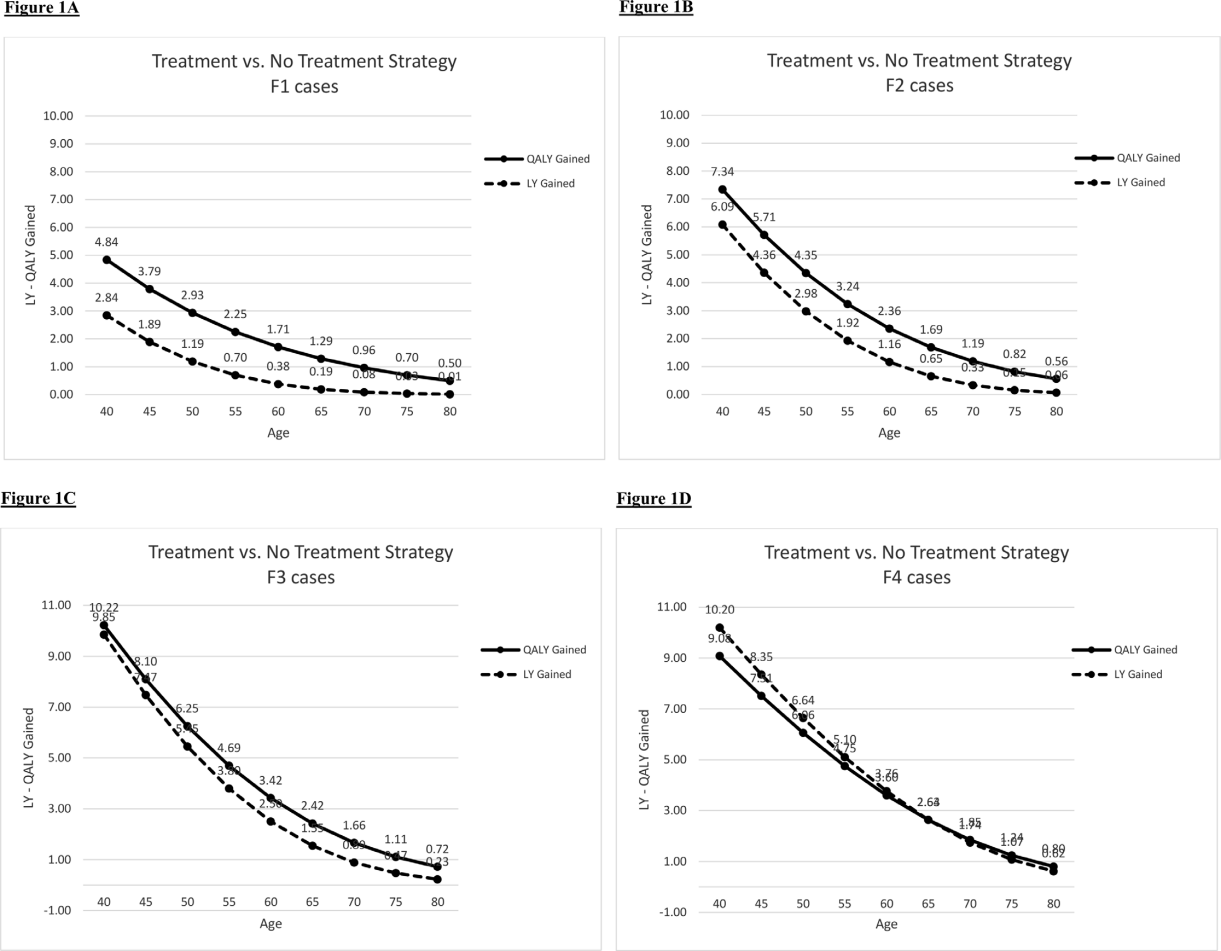
Life year (LY) and quality-adjusted life year (QALY) gained by age for fibrosis stages F1-F4 (Fig 1A-1D).

LY and QALY gained for treated *vs*. untreated HCV-infected patients were analyzed for each stage of fibrosis (F1 to F4) separately (Fig 1A–1D and Table 1). Generally, both the numbers of LY and QALY gained gradually decreased with advancing age but the rate of decline was slower with more advanced fibrosis level. In those patients with fibrosis stage F1, LY gained dropped below six months if treated by the age of 55, whereas for stages F2 and F3 LY gained was about six months if treated by the age of 65 or 70 years of age. A patient with fibrosis stage F4 gained more than one LY if treated for HCV by the age of 75. The QALYs gained for treated over untreated elderly were reasonably high even for those treated at an early fibrosis stage. As can be seen in Fig 1D, the curves of the LY and of the QALY merge for fibrosis stage F4, reflecting the diminished quality of life for patients with liver cirrhosis.

A Monte-Carlo simulation of 10,000 patients for projected rates of HCC and of liver transplantation in treated *vs*. untreated HCV patients is depicted in Tables 2 and 3, respectively. The rates are stratified by fibrosis stages F2, F3, F4 and by age, 50 or 70. As expected, the rates of HCC and of liver transplantation are higher with more advanced fibrosis stage. The rates are also higher in the 50-year-old group compared with the 70-year-old group, given the difference in life expectancy. Those patients treated for HCV demonstrate a significant decline in the rates of both HCC and liver transplantation. Nevertheless, the robustness of the reduction of the rate of both HCC and liver transplantation by anti-HCV treatment is diminished to a great extent among patients at the cirrhotic stage. However, among both the older and the younger patients, the extent to which complications are prevented in the treated population compared with the untreated population is similar.

**Table 2:** Monte-Carlo simulation of 10,000 patients (mean±SD); Rate of hepatocellular carcinoma (HCC).

		Rate of HCC	
METAVIR	Age	No Treatment	Treatment
**F2**	**50**	37.1% ± 16.5%	8.8% ± 0.8%
	**70**	21.0% ± 4.6%	4.5% ± 0.3%
**F3**	**50**	42.8% ± 24.1%	8.7% ± 0.8%
	**70**	30.3% ± 10.2%	5.7% ± 0.4%
**F4**	**50**	48.6% ± 38.5%	39.9% ± 19.9%
	**70**	42.2% ± 23.2%	32.4% ± 11.9%

**Table 3:** Monte-Carlo simulation of 10,000 patients (mean±SD); Rate of liver transplantation.

		Rate of Liver Transplantation	
METAVIR	Age	No Treatment	Treatment
**F2**	**50**	17.4% ± 3.1%	3.6% ± 0.13%
	**70**	8.6% ± 0.74%	2.0% ± 0.04%
**F3**	**50**	22.5% ± 5.3%	4.1% ± 0.17%
	**70**	13.3% ± 1.8%	2.8% ± 0.08%
**F4**	**50**	30.6% ± 10.5%	22.5% ± 5.3%
	**70**	22.3% ± 5.2%	16.2% ± 2.7%

### Sensitivity analysis

**Fig 2:**
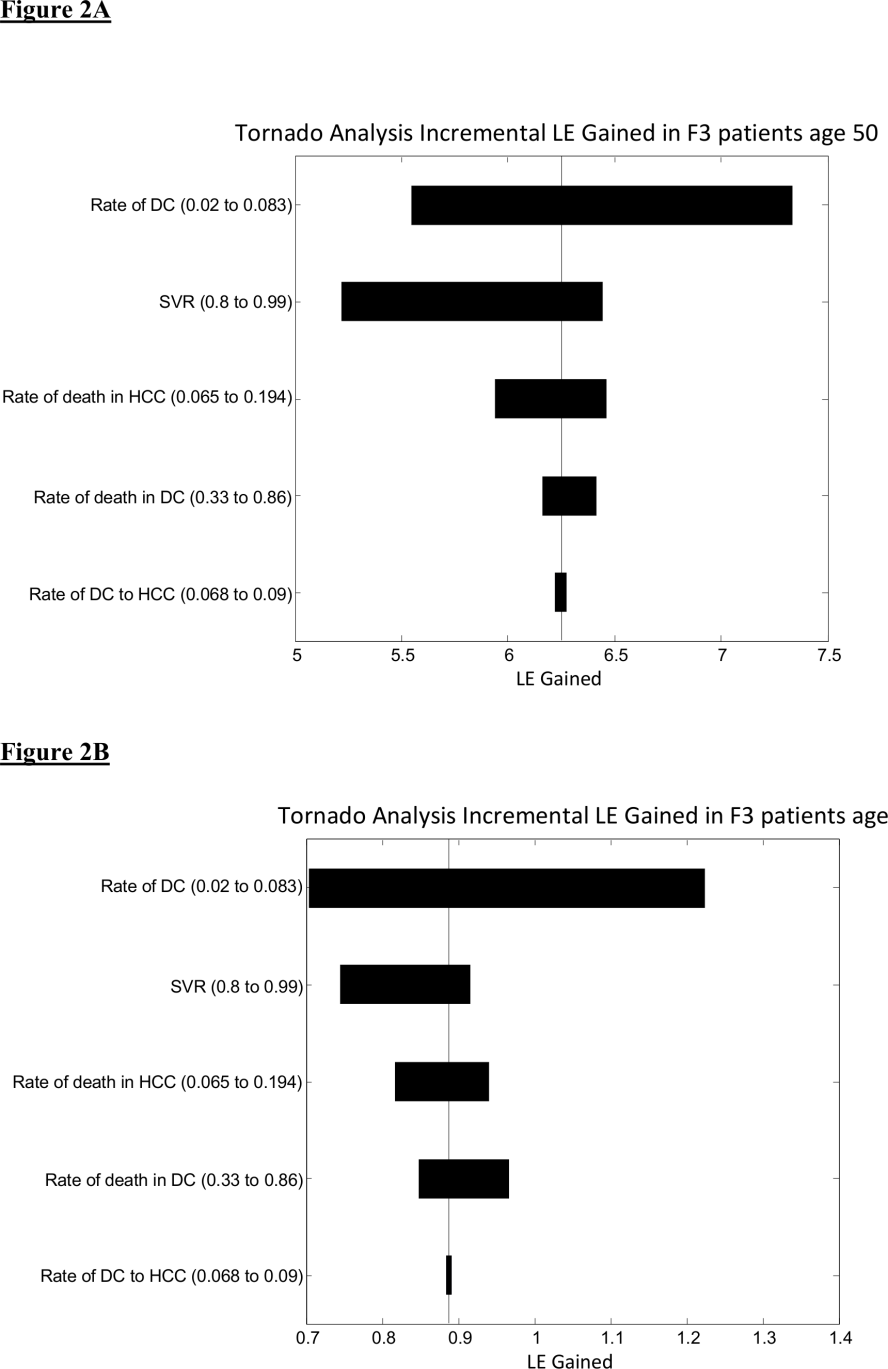
Tornado analysis for incremental life expectancy (LE) gained in F3 patients age 50 (Fig 2A) and 70 (Fig 2B).

Tornado sensitivity analysis was performed looking at several variables (Fig 2A and 2B). The increment in life expectancy gained among patients with F3 fibrosis stage aged 50 was compared with that among patients aged 70. The more significant increment in LE gained derived from improvement of SVR in the younger population. Increases in SVR rates from 80% to 99% for 50-year-olds, resulted in an increment of over one year of LE gained. With the same improvement in response in 70-year-olds, the survival benefit was only about two months.

## Discussion

In this study we found a significant life expectancy benefit of HCV treatment in elderly patients with advanced stages of fibrosis that decreased with patient's age. Patients with fibrosis stages F2 and F3 met our threshold of a 6-month gain in LY if treated by the age of 65 and 70 years, respectively, and patients with cirrhosis had a gain of at least one LY if treated by the age of 75.

There are some limitations to our study. Data from previous studies [17–19] demonstrate a more rapid fibrosis progression rate for those over 50 than for those younger than 50. Data regarding the rate of fibrosis progression for HCV patients older than 65 years are scarce. Therefore, our assumptions for patients older than 65 may lack accuracy and this may affect the predictive value of the model, notwithstanding Zhou et al [28], who reported that variation in rates of fibrosis progression had only a minimal impact on life expectancy gains. Also, our model assumed similar baseline characteristics for treated and untreated patients, though real-life experience clearly shows that untreated patients differ from treated patients on many demographic and clinical parameters.

Several large studies have documented an overall survival benefit for those patients with HCV who attain an SVR. A recent international, multicenter, longitudinal study with a long follow-up duration [11] showed SVR to be associated with prolonged overall survival and demonstrated a lower risk for all-cause mortality in patients with chronic HCV infection and advanced hepatic fibrosis who achieved SVR. The risk of all-cause mortality for patients without SVR was almost four times higher than that for patients with SVR. In another study of the association of SVR with all-cause death and liver transplantation as a combined end point among patients with advanced fibrosis or cirrhosis [12, 13], the adjusted cumulative proportion of patients who died or underwent liver transplantation after 7.5 years of follow-up was higher in patients not responding to peg-interferon and ribavirin therapy (27.2%) compared to patients with virological relapse (4.4%) or who achieved an SVR (2.2%). The largest study reported in the literature, which followed up a predominantly male population of U.S. veterans with all stages of liver fibrosis for a median of 3.8 years, reported 5-year mortality rates of 6.7% to 8.0% in patients with SVR *vs*. rates of 14.4% to 24.4% in patients without SVR [14]. The fact that these studies, although encouraging, had patients in their forties and fifties as their target population imposes serious limitations on our ability to extrapolate their conclusions to older patients.

Zhou et al [28] concluded from their decision analysis of a population stratified into five age groups from 60 to 80 treated with first-generation protease inhibitor combinations that the greatest life expectancy benefit was for treatment of younger patients with higher levels of fibrosis. Assuming an SVR rate of 70%, the mean life expectancy gained across all ages and stages of fibrosis was 2.18 years for women and 2.95 years for men, and all cohorts with fibrosis stage F2 and above reached a 6-month threshold of life expectancy gained. By and large these results concur with our analysis. Our analysis, however, was based on the results of more effective DAA regimens and included patients aged 40 to 80, thus enabling comparison of younger and older age groups.

Our analysis found additional advantages to HCV treatment as it resulted in a significant reduction in two of the major complications of advanced liver disease, namely HCC and the need for liver transplantation. However, unlike the advantage in life expectancy observed particularly in cirrhotic patients treated for HCV, reduction in both HCC and liver transplantation rates were more robust in those patients who had less advanced fibrosis (stages F2 and F3) than in the cirrhotic stage HCV. These observations are somewhat in conflict with the findings of van der Meer, et al [12], who reported in their long-term follow-up of patients with advanced fibrosis (F3-F4) that the risk of liver-related mortality or liver transplantation was negligible in those patients who attained an SVR compared with those who did not respond to anti-viral treatment, while the risk of HCC did not diminish completely even following successful treatment.

Our sensitivity analysis clearly demonstrated that higher SVR rates, which may be achieved with the new DAAs, will mainly affect the life expectancy of young rather than elderly patients. Zhou et al [28] also concluded that the older cohort did not realize substantial improvement in life expectancy gain despite the up to 90% increase in SVR. As the new interferon-free DAA regimens show a high safety profile, but carry a significant financial burden, one of the major considerations in the treatment of elderly patients is cost-effectiveness individualized to the patient's general health. Unlike the patients in clinical trials, more than 50% of older adults have three or more chronic diseases [29]. Thus, evidence-based clinical guidelines, which mainly focus on the management of a single disease, cannot be easily applied to adults with multimorbidity.

**Fig 3:**
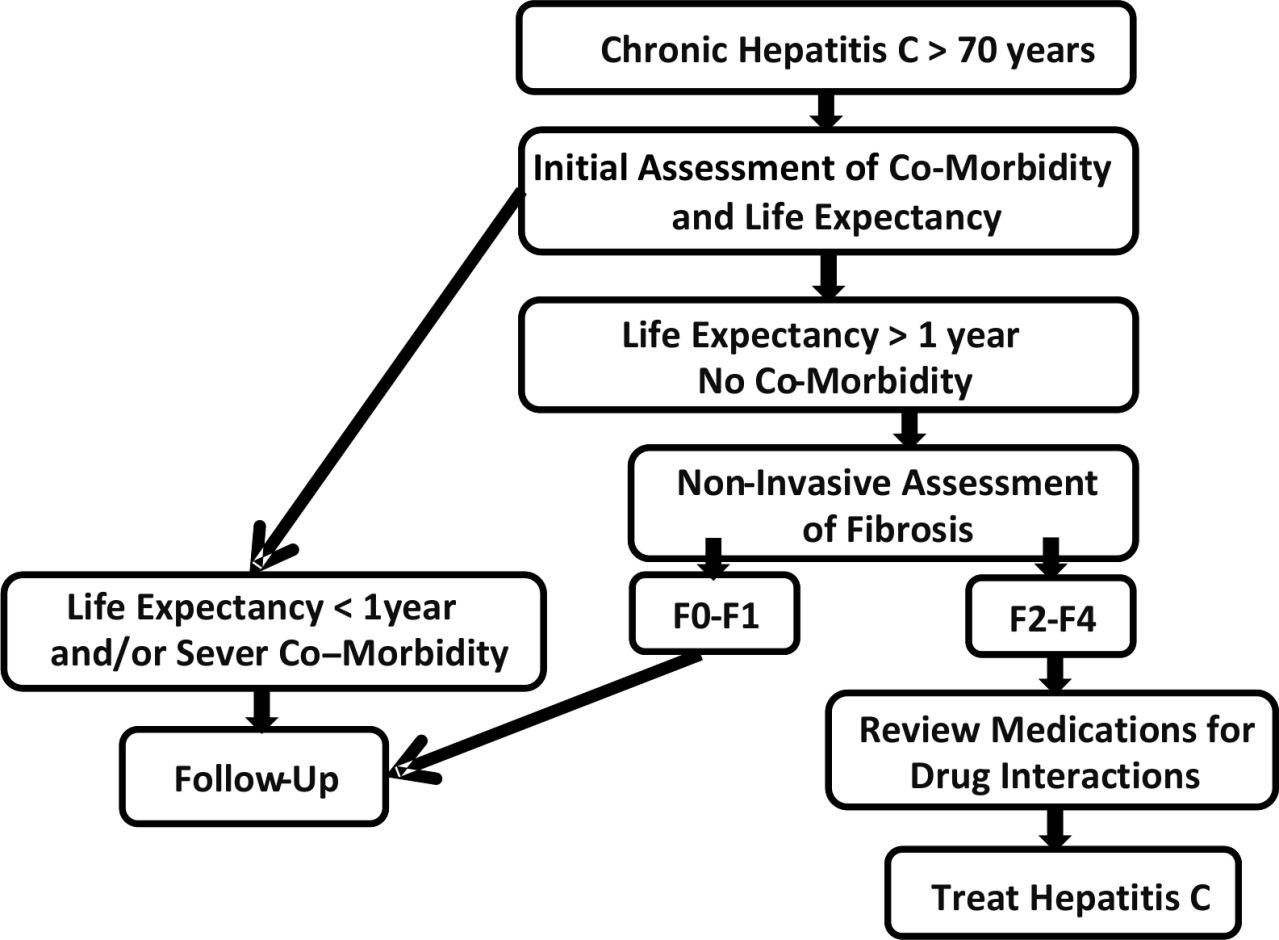
Suggested approach for treating HCV in the population over the age of 70.

Since currently complications of HCV mostly affect members of the elderly population, they are in urgent need of effective HCV treatment. The approach we suggest for the treatment of patients over 70 with chronic HCV is illustrated in Fig 3. For those patients who have no major co-morbidities, more than moderate fibrosis, and a life expectancy greater than one year, there is a possibility of offering treatment. This needs to be presented to the patient and discussed before a final decision.

Our report provides evidence supporting the consideration of HCV treatment in clinical practice for older patients with significant fibrosis, especially as shorter regimens with higher SVR rates and less adverse effects are becoming the standard-of-care for HCV infection. At the same time, medications that are already licensed, and those in development, need to be systematically tested for the aging populations. This will require an investment in the design, development and execution of specific clinical trials and in addition reporting of real world experience of the currently approved medication.

## Supporting Information

S1 File Tables. **Table A**: Assumption used for the Markov's Model. **Table B**: Life Expectancy for treated *vs*. non-treated patients by age for F1 to F4 (1–4).
(DOCX)

## Abbreviations

HCV: Hepatitis C virus
HCC: hepatocellular carcinoma
CDC: U.S. Centers for Disease Control and Prevention
USPSTF: U.S. Preventive Services Task Force
DAAs: direct-acting antivirals
SVR: sustained virological response
LY: life year
QALY: quality-adjusted life year
LE: life expectancy
