# Supplementary material for: Treatment of Chronic Hepatitis C in the Aged – Does It Impact Life Expectancy? A Decision Analysis
Source: PLoS One. 2016 Jul 13;11(7):e0157832. doi: 10.1371/journal.pone.0157832 (PMC4943667; doi:10.1371/journal.pone.0157832)
Supplement: S1 File — Table A: Assumption used for the Markov's Model. Table B: Life Expectancy for treated vs. non-treated patients by age for F1 to F4 (1–4). (DOCX) [file pone.0157832.s001.docx]

**Supporting Information Captions**

**Table A**: Assumption used for the Markov's Model

| **Description** | **Value** |
| --- | --- |
| Age | 50 |
| Cycle length (years) | 1 |
| SVR in F1 | 0.96 |
| SVR in F2 | 0.96 |
| SVR in F3 | 0.96 |
| SVR in F4 | 0.92 |
| **Transition Probabilities** |  |
| From F0 to F1 | 0.076 |
| From F1 to F2 | 0.070 |
| From F2 to F3 | 0.105 |
| From F3 to F4 | 0.050 |
| From F3 to HCC | 0.002 |
| From F4 to HCC | 0.017 |
| From DC to HCC | 0.076 |
| From F4 to DC | 0.038 |
| Annual death rate after LT | 0.050 |
| Annual death rate in DC | 0.129 |
| Annual death rate in HCC | 0.427 |
| LT mortality rate | 0.14 |
| Probability of LT in F4 patients | 0.050 |
| Probability of reverse F4 after SVR | 0.500 |
| Time horizon until age | 99 |
| **Utilities Values** |  |
| Utility of s/p LT | 0.82 |
| Utility of DC | 0.69 |
| Utility of F4 | 0.76 |
| Utility of HCC | 0.67 |
| Utility of F0 to F3 | 0.79 |
| Utility after SVR | 0.86 |

SVR=Sustained virological response; HCC=Hepatocellular carcinoma;

DC=Decompensated cirrhosis; LT=Liver transplantation

**Table B**: Life Expectancy for treated *vs*. non-treated patients by age for

F1 to F4 (1-4)

**Table B1**

| **Age** | **No Treatment (LY)** | **Treated (LY)** | **LY Gained** |
| --- | --- | --- | --- |
| **40** | 75.186 | 78.031 | 2.84 |
| **45** | 76.520 | 78.409 | 1.89 |
| **50** | 77.706 | 78.891 | 1.19 |
| **55** | 78.831 | 79.528 | 0.70 |
| **60** | 79.991 | 80.370 | 0.38 |
| **65** | 81.287 | 81.475 | 0.19 |
| **70** | 82.823 | 82.908 | 0.08 |
| **75** | 84.708 | 84.742 | 0.03 |
| **80** | 87.046 | 87.058 | 0.01 |

**Table B2**

| **Age** | **Treated (LY)** | **No Treatment (LY)** | **LY gained** |
| --- | --- | --- | --- |
| **40** | 71.810 | 77.897 | 6.09 |
| **45** | 73.945 | 78.306 | 4.36 |
| **50** | 75.837 | 78.817 | 2.98 |
| **55** | 77.553 | 79.477 | 1.92 |
| **60** | 79.174 | 80.337 | 1.16 |
| **65** | 80.805 | 81.456 | 0.65 |
| **70** | 82.565 | 82.898 | 0.33 |
| **75** | 84.583 | 84.737 | 0.15 |
| **80** | 86.992 | 87.056 | 0.06 |

**Table B3**

| **Age** | **Treated (LY)** | **No Treatment (LY)** | **LY gained** |
| --- | --- | --- | --- |
| **40** | 67.891 | 77.743 | 9.85 |
| **45** | 70.705 | 78.180 | 7.47 |
| **50** | 73.263 | 78.717 | 5.45 |
| **55** | 75.603 | 79.401 | 3.80 |
| **60** | 77.778 | 80.283 | 2.50 |
| **65** | 79.872 | 81.420 | 1.55 |
| **70** | 81.988 | 82.876 | 0.89 |
| **75** | 84.256 | 84.724 | 0.47 |
| **80** | 86.823 | 87.050 | 0.23 |

**Table B4**

| **Age** | **Treated (LY)** | **No Treatment (LY)** | **LY gained** |
| --- | --- | --- | --- |
| **40** | 57.842 | 68.043 | 10.20 |
| **45** | 61.966 | 70.317 | 8.35 |
| **50** | 65.900 | 72.542 | 6.64 |
| **55** | 69.630 | 74.734 | 5.10 |
| **60** | 73.155 | 76.918 | 3.76 |
| **65** | 76.491 | 79.129 | 2.64 |
| **70** | 79.679 | 81.421 | 1.74 |
| **75** | 82.800 | 83.874 | 1.07 |
| **80** | 85.981 | 86.596 | 0.62 |
